# *ICECREAM*: high-fidelity equivariant cryo-electron tomography

**DOI:** 10.1107/S2059798326001622

**Published:** 2026-03-27

**Authors:** Vinith Kishore, Valentin Debarnot, Ricardo D. Righetto, Benjamin D. Engel, Ivan Dokmanić

**Affiliations:** a Department of Mathematics and Computer Science, University of Basel, 4051Basel, Switzerland; b INSA-Lyon, Université Claude Bernard Lyon 1, CNRS, Inserm, CREATIS UMR 5220, U1294, Lyon, France; c Biozentrum, University of Basel, 4056Basel, Switzerland; Princeton University, USA

**Keywords:** cryogenic electron tomography, self-supervised learning, machine learning, tomography, deep learning

## Abstract

Cryo-electron tomography (cryo-ET) visualizes 3D cellular architecture in its near-native state. The various deep-learning methods have improved denoising and artifact correction, but remain challenged by a very low signal-to-noise ratio, a restricted tilt range (‘missing wedge’) and the lack of ground truth. Here, we present *ICECREAM*, which bridges earlier self-supervised methods with the recent equivariant imaging framework [Chen *et al.* (2021 ▸), *IEEE/CVF International Conference on Computer Vision (ICCV)*, pp. 4359–4368]. Across diverse experimental datasets, *ICECREAM* achieves substantially better denoising and more reliable missing-wedge filling than existing methods. *ICECREAM* can be applied to any tomography problem that provides two statistically independent views of the volume; in cryo-ET these are obtained by dose splitting or angular partitioning of the tilt series. *ICECREAM* is openly available at https://github.com/swing-research/icecream.

## Abstract 2

We present *ICECREAM*, a self-supervised approach that achieves substantially better denoising and more reliable missing-wedge recovery in cryo-ET, while reducing training and inference time relative to comparable baselines.

## 1. Introduction

Cryo-electron tomography (cryo-ET) has become a central technique in structural biology, enabling the direct visualization of macromolecular assemblies in their native cellular environment. Its ability to provide three-dimensional structural information at nanometre resolution without the need for crystallization has made it an essential tool for studying complex and heterogeneous biological systems such as organelles, pathogens and large protein complexes. Cryo-ET complements single-particle cryo-EM by offering spatial context and capturing the structural diversity of biological specimens *in situ* (Navarro, 2022 ▸; McCafferty *et al.*, 2024 ▸).

Despite its rapidly growing importance, cryo-ET remains extremely challenging from a computational and signal processing perspective. It involves imaging tiny, delicate, radiation-sensitive samples using high-energy electrons while tilting the sample with a mechanical stage. To minimize beam-induced damage, only a limited number of projections can be collected, each with a low dose. This results in a very low signal-to-noise ratio (SNR), with complex noise statistics that are difficult to model or filter without distorting meaningful signal (Vulović *et al.*, 2013 ▸).

Further problems arise from mechanical limitations and sample geometry which prevent imaging across the full ±90° tilt range, leaving a significant portion of the Fourier space unobserved. This so-called *missing wedge* results in anisotropic artifacts such as streaking and smearing that degrade the resolution of reconstructed volumes (Shkolnisky & Singer, 2012 ▸; Chen *et al.*, 2016 ▸).

A variety of post-processing and reconstruction techniques have been proposed to address these challenges. Classical approaches include filtering (Feldkamp *et al.*, 1984 ▸; Heide *et al.*, 2007 ▸; Frangakis, 2021 ▸), subtomogram averaging (Tegunov *et al.*, 2021 ▸; Burt *et al.*, 2024 ▸) and model-based regularization (Gilbert, 1972 ▸; Andersen & Kak, 1984 ▸; Deng *et al.*, 2016 ▸; Yan *et al.*, 2019 ▸). More recently, deep learning has emerged as a powerful tool for improving the visual quality of the reconstructed volumes, particularly in denoising and missing-wedge artifact removal.

### 1.1. Self-supervised learning

Our proposed method, *ICECREAM*, is self-supervised, meaning that it does not require noiseless and artifact-free volumes to train. Self-supervised approaches are among the strongest successful applications of deep learning in cryo-ET reconstruction, where ground-truth data do not exist. Perhaps the most common is *cryoCARE* (Buchholz *et al.*, 2019 ▸), which builds on the more general *Noise*2*Noise* principle (Lehtinen *et al.*, 2018 ▸): leveraging independent noisy observations to train a denoising neural network without clean supervision.

Self-supervised methods have also been developed for missing-wedge correction. *IsoNet* exploits the fact that the orientation of the missing wedge in Fourier space does not depend on the orientation of the volume (Liu *et al.*, 2022 ▸). By training on rotated versions of the input subtomograms, *IsoNet* can partially fill the missing spectral regions. While it also includes a denoising mechanism, practitioners often apply *cryoCARE* before passing to *IsoNet* as this significantly improves denoising. To address the separate denoising and missing-wedge completion, *DeepDeWedge* takes a more principled approach and combines *Noise*2*Noise *with the rotation-aware strategy used in *IsoNet* (Wiedemann & Heckel, 2024 ▸). This gives a unified framework at the cost of a considerably increased training time. A detailed comparison of our approach with *DeepDeWedge* and *cryoCARE*+*IsoNet* is provided in Appendix *A*[App appa].

### 1.2. Supervised learning

A drawback of self-supervised methods is that they need to be trained from scratch for each new acquisition. Despite several attempts to train such models on large-scale datasets, their performance degrades when applied to unseen samples or different acquisition conditions (Wiedemann & Heckel, 2024 ▸). To overcome this challenge, we recently introduced *CryoLithe* (Kishore *et al.*, 2025 ▸), the first supervised deep neural network designed to generalize across acquisitions, which eliminates the need for retraining. *CryoLithe* is robust to unseen observations thanks to localized learning strategies (Khorashadizadeh, Debarnot *et al.*, 2025 ▸; Khorashadizadeh, Liaudat *et al.*, 2025 ▸), but it still implicitly relies on self-supervised learning to generate high-quality training targets. Hence, improving the quality of self-supervised reconstruction methods remains crucial.

### 1.3. Group equivariance

Chen and coworkers (Chen *et al.*, 2021 ▸, 2022 ▸) introduced a theoretical framework for solving inverse problems by leveraging group equivariance as a form of regularization. When the group is the rotation group, this is similar to the property leveraged by *IsoNet* and *DeepDeWedge*. Indeed, most successful self-supervised methods in cryo-electron microscopy and tomography rely on augmenting the training dataset with rotated subtomograms and a ‘manual’ masking of the missing wedge (Liu *et al.*, 2022 ▸, 2025 ▸; Wiedemann & Heckel, 2024 ▸). Training on rotation-augmented data encourages the composition of the network with the forward operator to be rotation-equivariant, but the way this is achieved in prior work on cryo-ET is different from the equivariant imaging framework of Chen and coworkers. We note that there exist neural network architectures that are equivariant to given groups by design (Chaman & Dokmanić, 2021*a* ▸,*b* ▸; Herbreteau *et al.*, 2023 ▸), but the composition with a non-injective forward operator (as in missing-wedge cryo-ET) requires one to leverage the structure of the data.

### 1.4. Contributions and outline

In this paper, we connect equivariant imaging as introduced by Chen and coworkers (Chen *et al.*, 2021 ▸, 2022 ▸) with cryo-ET. The resulting method, *ICECREAM*, achieves state-of-the-art denoising and missing-wedge correction. We demonstrate *ICECREAM* on a variety of real experimental datasets with very diverse spatial statistics. We also optimize it so that it is faster and more memory efficient than existing self-supervised methods, while yielding better reconstructions.

The paper is organized as follows. Section 2 describes the acquisition model, the proposed method and its training procedure. Section 3 presents quantitative and visual results on real data. Section 4 discusses the implications and limitations of the proposed approach. Additional experiments and details of baseline methods are provided in the Appendices.

*ICECREAM* is openly available under the MIT License. The source code and the documentation can be found at https://github.com/swing-research/icecream.

## 2. Methods

We propose to learn a function *f*_ϕ_, here a deep neural network,with parameters 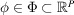, which maps noisy and missing-wedge-degraded subtomograms to volumes free of artifacts. To achieve this, we combine two consistency criteria that jointly constrain the network to perform effective denoising and missing-wedge correction: *Noise*2*Noise* and equivariance.

### 2.1. Acquisition model

Cryo-ET reconstruction algorithms estimate a three-dimensional (3D) volume model *V* from a set of aligned two-dimensional (2D) projections called a tilt series. Let 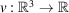 be the target volume, 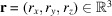 and *v*_θ_ be the volume rotated about the *y* axis by θ. Then 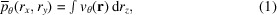
where θ ranges over the finite set of tilt angles Θ, typically between −60° and +60° in increments of 2° or 3°. The missing tilt angles lead to the so-called missing wedge in Fourier space.

We observe a noisy version of 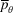, denoted by *p*_θ_, on a discrete pixel grid. (We keep continuous coordinates here to avoid technicalities related to interpolation and sampling theorems.) The tomographic inverse problem consists of computing an estimate 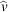 of *v* from the data 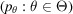. A classic approach is the filtered backprojection (FBP), which has a simple description in Fourier space^1^ (Kak & Slaney, 2001 ▸; Harauz & van Heel, 1986 ▸). By the Fourier slice theorem, the 2D Fourier transform of 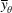 coincides with the slice of the 3D Fourier transform of *v* at angle θ or, equivalently, with the central slice of the 3D Fourier transform of *v*_θ_. Concretely, denoting Fourier transforms by uppercase letters (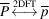) and using the convention
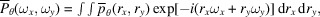
(and analogously for 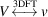, 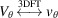
*etc.*), the Fourier slice theorem states that 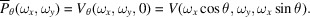
The FBP then takes the observed projections *p*_θ_ for all θ ∈ Θ, computes their Fourier transforms *P*_θ_ and places them along the corresponding planes with normal vectors 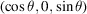 in the Fourier space of 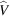 so that 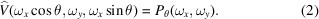
For this to make sense, we have to assume that Θ is a continuous interval of tilts; in practice, one has to invoke the sampling theorem and some form of interpolation.

By the Fourier slice theorem, it is immediate that without noise and with Θ the full angular range, equation (2) implies perfect reconstruction. When Θ is a subset of the full range, FBP fills the missing Fourier information with zeroes. It is convenient to define an operator which combines the forward operator (1) and the FBP reconstruction so that it maps volumes to volumes rather than volumes to projection stacks. Denoting the limited-view tomographic forward operator by *F*, the orthogonal projection *A* = *F*^+^*F*, where (·)^+^ is the Moore–Penrose pseudoinverse, zeroes out the Fourier components inside the missing wedge. It thus enacts the ‘missing-wedge corruption mechanism’ in the sense that *A*(*V*) is the version of *V* with the missing wedge zeroed out in Fourier space and the corresponding artifacts in real space.

The FBP formula is efficient and simple but the resulting volumes tend to be noisy, with pronounced missing-wedge artifacts. We use deep learning to improve them. Here, we follow the established self-supervised template: first use the FBP to compute two independent volumes, then use self-supervised learning (*Noise*2*Noise* and equivariance) on subtomograms to compute the final enhanced volume.

### 2.2. Working with subtomograms

Working with subtomograms is both a practical necessity, since the full tomograms are often too large to fit in memory together with deepnet activations, and a statistical necessity, since a single tomogram contains many subtomograms which can be used as training data. For illustration, a typical tomogram we work with is of dimension 928 × 928 × 464 voxels and we extract subtomograms of size 72 × 72 × 72.

This means that our neural network *f*_ϕ_ takes subtomograms as inputs and produces subtomograms as the output. As we show below, applying the equivariant learning principle while maintaining computational efficiency will require us to apply the missing-wedge corruption *A* to subtomograms. This cannot be performed exactly without knowing the entire volume. Indeed, the operator *A* acts on the entire volume: it is nonlocal and does not commute with cropping. Letting *C* be an operator which extracts a subtomogram from a larger tomogram by zeroing the rest, we have in general that *AC* ≠ *CA*. One way to see this is to note that *CA* first zeros out the missing wedge in the entire volume and then crops the sub­tomogram. Since cropping is a convolution with a sinc function in Fourier space, the resulting missing wedge will not be exactly empty but contain the tails of this sinc. By contrast, *AC* first crops the subtomogram and then zeros out the missing wedge in the cropped volume, which will be truly zeroed. As in earlier work, we ignore the resulting approximation error since applying *A* to subtomograms dramatically improves computational efficiency and seems (sufficiently) benign in practice.

### 2.3. Independent noisy observations

Self-supervised denoising methods such as *cryoCARE* (Buchholz *et al.*, 2019 ▸) leverage the availability of two independent noisy observations of the same underlying signal. In cryo-ET such independent realizations are available thanks to modern direct detectors which record multiple frames for every tilt angle (Faruqi & Henderson, 2007 ▸). These frames are then usually averaged after motion compensation in order to increase the signal-to-noise ratio. *CryoCARE* leverages the independence of noise realizations across the frames to outperform simple averaging. We follow this template and assume that for each subvolume 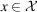, we obtain two independent observations 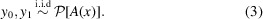
For simplicity, we model the noise by a random operator 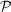 acting on the missing-wedge-corrupted subtomogram *A*(*x*).^2^ This includes a variety of possibly unknown noise types. Noise in cryo-ET is complex and arises from a combination of sources, including detector characteristics, sample-induced scattering, background signal and electronic noise. Due to this complexity, a precise parametric model of the noise seems intractable (Quinto *et al.*, 2009 ▸; Yang *et al.*, 2024 ▸).

We let 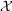 be the set of all subtomograms extracted from the (unknown) clean tomogram *v* and 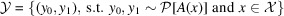
be the set of observations corresponding to 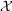.

In our implementation, we extract the subtomogram pairs 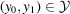 following *IsoNet*’s procedure (Liu *et al.*, 2022 ▸), after a tomogram-level normalization. This involves extracting the parts of the tomograms that are likely to contain structures of interest and avoiding the parts that contain only ice. Our implementation supports masks of interest areas provided by users, for example generated with *Slabify* (https://github.com/CellArchLab/slabify-et).

### 2.4. Denoising and data consistency

We first ensure that the reconstruction is consistent with the measurements. For this, we follow the *Noise*2*Noise* principle, which is among the most effective existing denoising techniques in cryo-ET. It amounts to minimizing the following cost function: 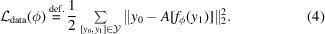
Notice that *y*_0_ and *y*_1_ can be swapped to augment the training dataset. This self-supervised loss can be related to its supervised counterpart. For completeness, we give a standard derivation in Appendix *B*[App appb].

### 2.5. Missing-wedge prediction

The data-fidelity loss enforces consistency only within the visible wedge, leaving the missing wedge largely unconstrained. To address this, we introduce an additional loss term based on rotation equivariance in the spirit of Chen *et al.* (2021 ▸). The proposed loss function integrates two complementary components. Firstly, it enforces equivariance of the composition of the reconstructor network *f*_ϕ_ and the operator *A*, *f*_ϕ_ ○ *A*, under 3D rotations. Secondly, it ensures reconstruction consistency between the two measurements *y*_0_ and *y*_1_ corresponding to the same subtomogram 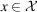. Together, these terms promote a consistent filling of the missing wedge.

Let *G* denote the finite set of 3D subtomogram rotations: the 20 rotations that can be applied without interpolation, together with their flips, excluding the identity and 180° rotations, so that |*G*| = 40. For *g* ∈ *G* let *R*_*g*_ be the corresponding transformation acting on volumes. We also define the rotated-wedge masking operator 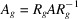
which corresponds to applying the missing-wedge mask in the orientation induced by *g*.^3^

We then define the equivariance loss as 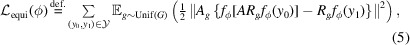
Intuitively, the equivariant loss is minimized if the neural network performs well at filling the missing wedge on any rotation of the input volume. The loss would ideally be computed by replacing *f*_ϕ_(*y*_0_) and *f*_ϕ_(*y*_1_) by the clean sub­tomogram *x*. Since this clean subtomogram is unavailable, we use the current network output as a ‘plug-in’ estimate. As in the denoising loss (equation 4), the roles of *y*_0_ and *y*_1_ can be swapped to augment the training data. The original equivariant imaging loss does not include the masking operator *A*_*g*_; we observe that using it gives slightly more detailed reconstructions; see Appendix *F*[App appf].

One key difference between *DeepDeWedge* and *ICECREAM* is the double application of *f* in equation (5), which leads to a simpler, principled training protocol; a detailed discussion is given in Section *A*1. Finally, we note that the rotation sampling procedure is almost identical to that of *IsoNet*, with the addition of flips to enhance data diversity.

### 2.6. Training

To obtain the final reconstructions, we minimize a total loss that combines the data-fidelity and equivariance terms, 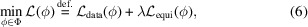
where λ > 0 is a regularization parameter to balance the importance of the two terms. We empirically found that λ = 2 provides good results, with strong denoising and without suppressing detail. An approximate solution of problem (6) is computed using automatic differentiation and the Adam optimizer (Kingma, 2014 ▸).

### 2.7. Inference

The final reconstruction 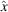 is computed by applying the trained neural network twice: first by computing the denoised tomograms and then by improving the missing-wedge correction, 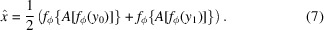
Applying the network only once leads to worse estimation of the missing wedge. This is because the equivariance loss, which is responsible for filling the missing wedge, is evaluated using the denoised volumes *f*_ϕ_(*y*_0_) and *f*_ϕ_(*y*_1_).

For optimal performance *f*_ϕ_ should be trained on sub­tomograms of a single tomogram and evaluated on these same subtomograms. This, however, requires training a separate network for each tomogram, which is time demanding. This process can be accelerated when reconstructing multiple tomograms with similar spatial statistics: often with similar biological content and acquired with the same microscope. As we show in the next section, in such cases it is possible to train a single network for all tomograms. This network can be effective even on tomograms not used for training as long as they are structurally sufficiently similar. When working with a structurally distinct tomogram, a network trained on multiple tomograms can still be used as a warm start for problem (6); we show this in Section 3.4.1.

## 3. Results

We test *ICECREAM* on a variety of real datasets. We train the model with a patch size of 72 × 72 × 72 voxels and a batch size of 8 on a GeForce RTX 4090 GPU with 24 GB memory using the Adam algorithm. We stop the training after 50 000 iterations when the marginal change in estimated subtomograms becomes insignificant. A more detailed analysis of *ICECREAM*’s training along iterations is given in Appendix *F*[App appf]. Further details of the architecture and the training parameters used for the experiments can be found on the GitHub page for the project. We compare *ICECREAM* with *DeepDeWedge*, *cryoCARE*+*IsoNet* and *CryoLithe*. *DeepDeWedge* and *IsoNet* v.0.3 have been trained using the code provided by their authors with the same patch size of 72 × 72 × 72, *DeepDeWedge* for 1000 epochs or a day, whichever limit is met first, and *IsoNet* for 30 epochs. *CryoLithe* gives results of similar quality as *cryoCARE*+*IsoNet*, while being much easier to use and much faster as it does not require training a neural network. As a consequence, it can perform better than *cryoCARE*+*IsoNet* on tomograms that are difficult to process. For a tomogram of size 928 × 928 × 464, which is more or less the size of all the tomograms processed in this paper, *ICECREAM* is trained for 12 h. On the same tomogram size, *DeepDeWedge* usually trains for 24 h and *cryoCARE*+*IsoNet* for 6 h.

When the tilt-series frames can be split to reconstruct four statistically independent tomograms, we train two independent models (*ICECREAM*, *DeepDeWedge* or *cryoCARE*+*IsoNet*), each using two tomograms, and quantitatively assess the results using the Fourier shell correlation (FSC) between the two estimated tomograms. We emphasize that this technique *a priori* measures how consistent or how stable to noise an algorithm is, rather than the resolution of the reconstruction. Stability is nonetheless a desirable property of inverse problem solvers.

One major downside of self-supervised algorithms, and in particular of *DeepDeWedge*, is its long training time, on the order of dozens of hours. A direct implementation of *ICECREAM* suffers from the same drawback. To speed it up, we used mixed-precision arithmetic on the GPU, storing the various quantities in half-precision (float 16). This significantly reduces the training time, without damaging the reconstruction performance. In the following, all *ICECREAM* results have been obtained using mixed precision.

### 3.1. Denoising and missing-wedge correction of diverse cryo-ET datasets

**Figure 1:**
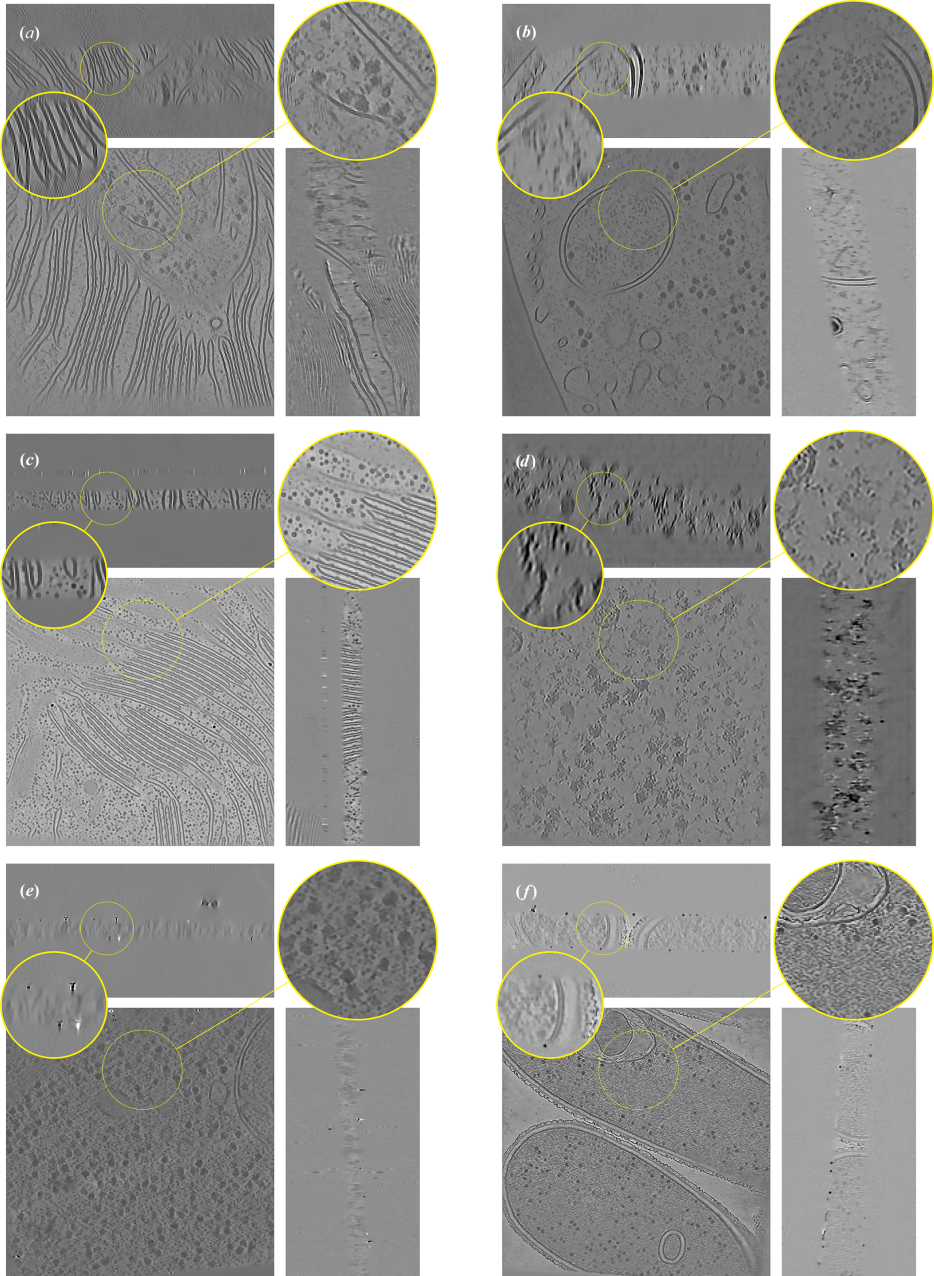
*ICECREAM* applied to a variety of tomograms, from different microscopes, different resolutions and different tilt schemes. Information about the tomograms is reported in Table 1 ▸. Corresponding FBP and *DeepDeWedge* reconstructions are displayed in Figs. 9 ▸ and 10 ▸, respectively.

We first showcase *ICECREAM* by reconstructing a variety of cryo-ET tomograms with different biological content and coming from different microscopes. The results are presented in Fig. 1 ▸, with additional details in Table 1 ▸. All tomograms have been downsampled by a factor of 4. *ICECREAM* performs equally well on different tomograms despite great structural differences. We observe stronger denoising and missing-wedge correction on tomograms that have been obtained with well aligned tilt series; see Fig. 1 ▸(*f*). For comparison, Fig. 1 ▸(*e*) shows a tomogram obtained from visually less accurately aligned tilt series and hence somewhat lower quality. The corresponding FBP and *DeepDeWedge* reconstructions are shown in Figs. 9 and 10 (Appendix *C*[App appc]). On the six tomograms, *ICECREAM* produces significantly sharper and better denoised structures in the *XY* plane compared with *DeepDeWedge*. In the *XZ* and *YZ* planes, *ICECREAM* generally estimates membranes and particles with improved contrast and sharper definition, although in some cases the improvement is limited, as with the tomogram in Fig. 1 ▸(*e*) which suffers from less careful pre-processing. Note that we ‘reverse cherry-picked’ Fig. 1 ▸(*e*) to illustrate the limitations of *ICECREAM*. On the great majority of the many tomograms we tested, *ICECREAM* clearly outperforms the baselines. A side-by-side comparison between *ICECREAM* and the reference methods is presented in the next sections.

### 3.2. Post-processing of *Thermoanaerobacter kivui*tomograms

**Figure 2:**
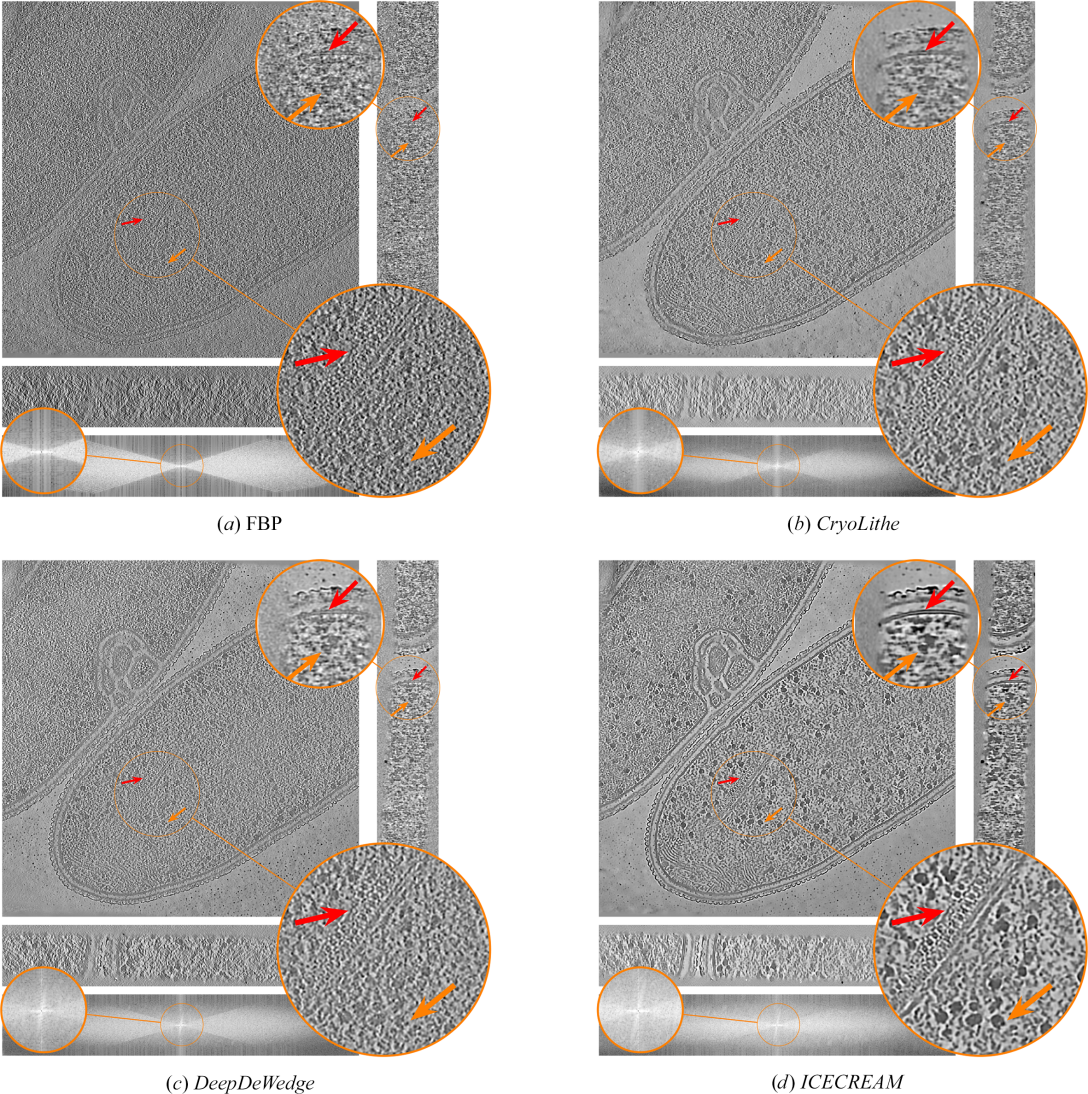
Orthogonal slices of the *T. kivui* (EMPIAR-11058) reconstruction using state-of-the-art denoising and missing-wedge correction methods. Additionally, we show the *XZ* slice of the Fourier transforms of the corresponding reconstructions on a log-magnitude scale, where the observed and missing-wedge supports are visible. *ICECREAM* performs better denoising, as visible in the background, and yields sharper detail, as indicated by the arrows. In the Fourier domain, *ICECREAM* shows stronger signal in the missing-wedge region (brighter areas) compared with other methods.

Next, we visually compare our proposed approach with the baseline methods on *T. kivui*, an anaerobic bacterium that efficiently fixates carbon (Dietrich *et al.*, 2022 ▸). The raw dataset is available from the Electron Microscopy Public Image Archive (EMPIAR-11058). Results are shown in Fig. 2 ▸. We see that *ICECREAM* gives better background denoising than the baselines, with empty regions appearing noticeably cleaner. It also makes it easier to discern small structures (for example the orange arrow) and better estimate high-frequency patterns (for example the red arrow). This shows the potential of *ICECREAM* to facilitate manual or automated particle picking as a first step for subtomogram averaging.

### 3.3. Influence of the splitting strategy

*ICECREAM* and the self-supervised baselines all require the input data to be split. The preferred way to do this is by splitting the dose to obtain two tilt series with independent noise. In some situations, however, for example with older datasets, this is not possible. An alternative is then to split a single tilt series along the tilt angles, although this reduces the Fourier space sampling density. In the following, we quantitatively evaluate the impact of the splitting strategy on the different algorithms using the FSC metric.

We work with the dataset for flagella of *Chlamydomonas reinhardtii*, which is the tutorial dataset used in *cryoCARE* and contains ten raw frames per tilt. The projections were collected at angles from −65° to +65° with 2° increments; the pixel size is 2.36 Å. The tilt series is further downsampled by a factor of 6, resulting in an effective pixel size of 14.16 Å.

**Figure 3:**
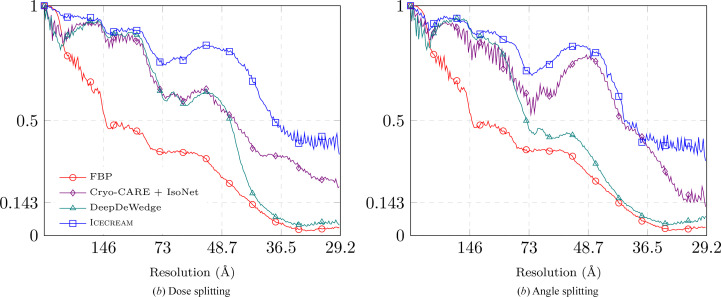
FSC of different methods on the dataset of *C. reinhardtii* flagella. FSC was computed by splitting the data into four realizations, from which two pairs were used to produce two independent tomograms. *ICECREAM* performs better than baselines uniformly at all frequencies and for both splitting strategies.

For the dose-splitting evaluation, we use two frames per projection and average them at inference time to construct the tilt series. Frames 9 and 10 are excluded so that the four resulting tilt series have comparable SNRs, assuming that all frames contribute equally. We run *DeepDeWedge*, *cryoCARE*+*IsoNet* and *ICECREAM* independently on sets 1–2 and 3–4, obtaining two reconstructions. The two post-processed tomograms are then compared using FSC, with the results reported in Fig. 3 ▸(*a*).

For angle splitting, we first average the four frames separately to obtain two tilt series, again excluding frames 9 and 10 to have the same amount of information as for dose splitting. Each tilt series is further split along tilt angles and fed into *DeepDeWedge*, *cryoCARE*+*IsoNet* and *ICECREAM*. As before, we obtain two independent reconstructions and evaluate them using FSC; see Fig. 3 ▸(*b*).

**Figure 4:**
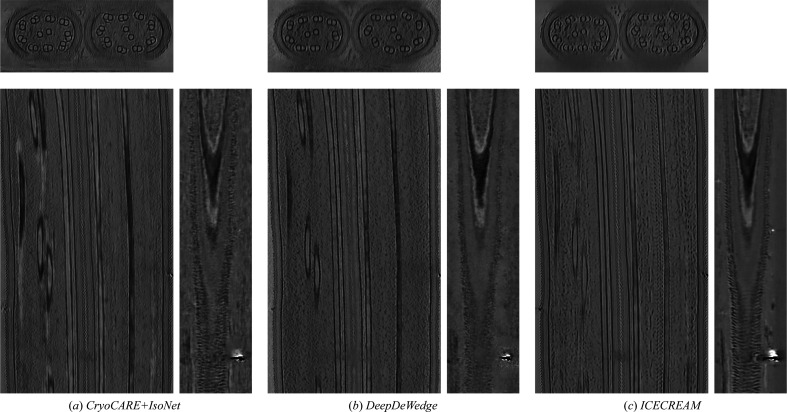
Orthogonal slices of a *C. reinhardtii* flagella tomogram estimated using dose splitting. The volumes have been cropped to remove empty areas.

**Figure 5:**
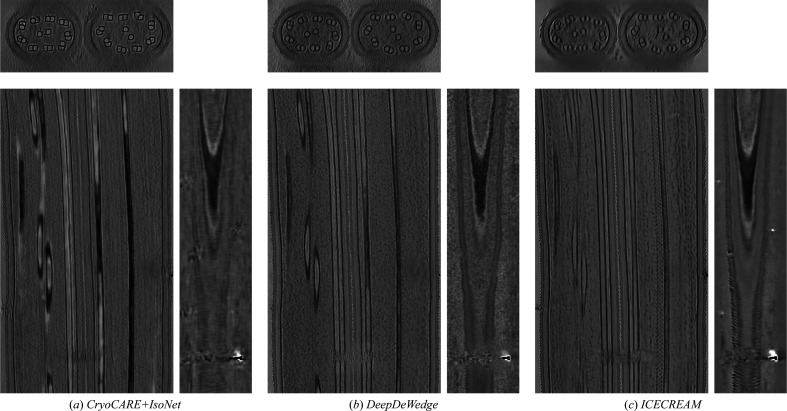
Orthogonal slices of a *C. reinhardtii* flagella tomogram estimated using angle splitting. The volumes have been cropped to remove empty areas.

*ICECREAM* outperforms the baselines for both splitting strategies. It is remarkable that the FSC for *ICECREAM* remains high even at high frequencies. As noted earlier, this should interpreted with some care. What it shows is that the two statistically independent reconstructions produced by *ICECREAM* are in better agreement than for other algorithms; put differently, *ICECREAM* is more stable to perturbations. This is likely in part a consequence of strong background denoising where other algorithms take a high-frequency FSC hit. This is desirable: an effective method should remove noise in a consistent way across independent inputs. The corresponding processed tomograms are shown in Fig. 4 ▸ (dose splitting) and Fig. 5 ▸ (tilt splitting). Notice that *DeepDeWedge* seems to attenuate the effect of the missing wedge slightly better than *ICECREAM*, for example in Fig. 5 ▸. We emphasize, however, that in order to compute the FSC curves, each tilt of the tilt series contains only two out of ten frames, which significantly reduces the SNR per tilt and, consequently, the training tomograms. It is not a splitting strategy that is used in practice. On full tilt series, we generally observe visually similar or better reconstructions using *ICECREAM*; see Figs. 1 ▸ and 10.

Comparing Fig. 3 ▸(*a*) and Fig. 3 ▸(*b*), dose splitting leads to a slightly better performance. This result mirrors the conclusion of the *cryoCARE* paper that dose splitting should be preferred (Buchholz *et al.*, 2019 ▸). In our case, the difference is relatively small; see Fig. 12 (Appendix *D*[App appd]) for a side-by-side comparison for *ICECREAM*.

### 3.4. Performance of self-supervised methods on unseen data

One of the main limitations of existing self-supervised post-processing methods is that they require hours of training for each single tomogram. Here, we explore using a network trained on a fixed dataset to process new tomograms. We show that such a network can be used without re-training if applied to data similar to those it was originally trained on. If the tomogram to process is substantially different, a pre-trained network can still be used to speed up training as it only needs to be fine-tuned for the new tomogram.

**Figure 6:**
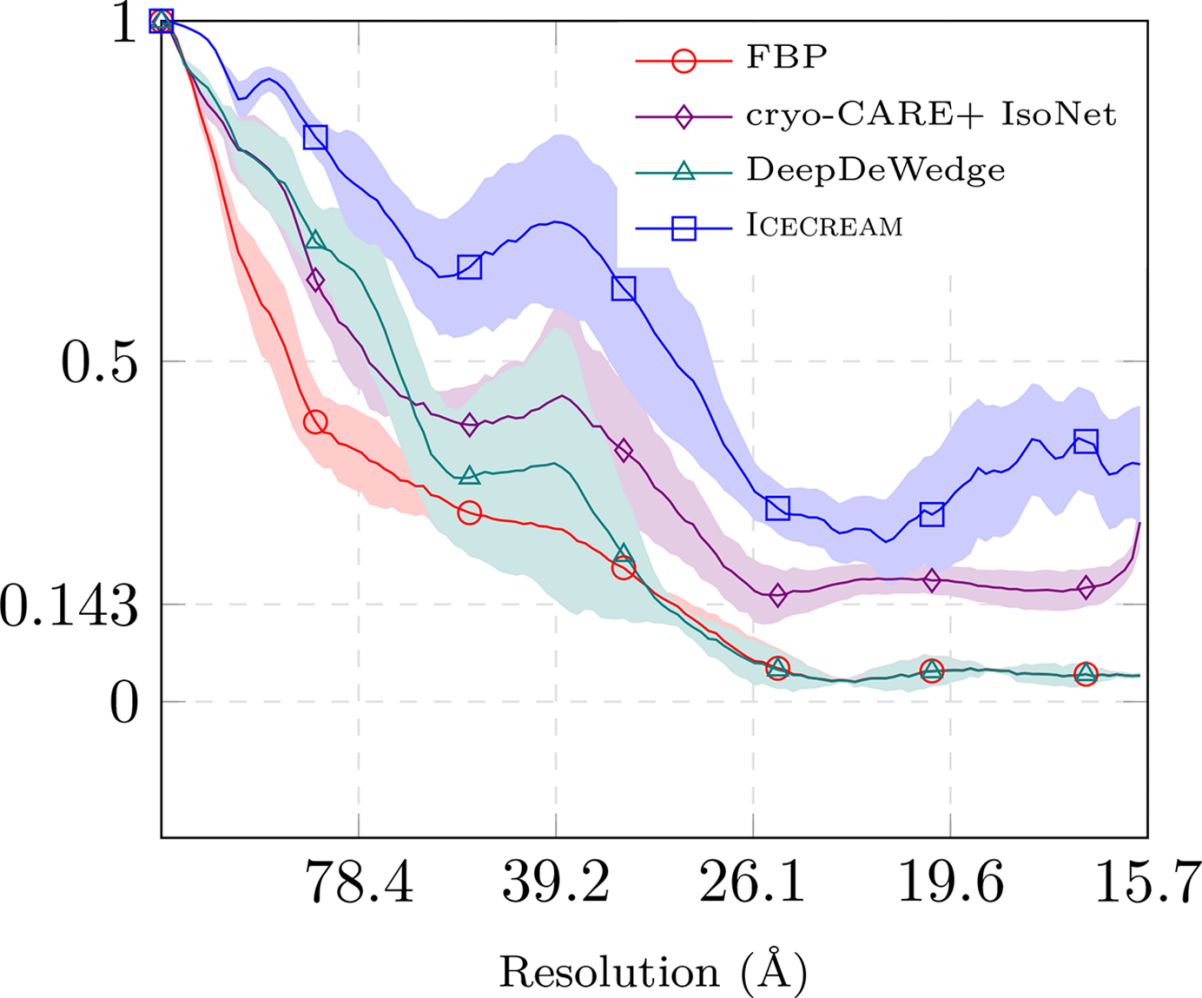
Out-of-distribution performance of self-supervised methods. The reconstructed volumes were obtained by using a neural network that has been trained on four different tomograms coming from the same microscope and containing similar biological content to the test tomograms. The FSC curve of *ICECREAM* consistently improves over FBP and improves over *cryoCARE*+*IsoNet* and *DeepDeWedge* throughout the spectrum. The plain curves correspond to the mean FSC value, and the shaded areas to the standard deviation around the mean value, computed on ten tomograms.

We selected a subset of the EMPIAR-11830 dataset, which contains approximately 2000 tilt series of *C. reinhardtii*. From this dataset, we chose ten tilt series for training and four for testing, all at bin 4, resulting in a pixel size of 7.84 Å. The dataset provides dose-fractionated ODD and EVEN tilt series. We used *IMOD* to generate the corresponding FBP reconstructions from the tilt series that serve as inputs. During training, both ODD and EVEN volumes were used. However, at inference, we reconstructed the ODD and EVEN volumes separately on the test set. We then evaluated the FSC between the ODD and EVEN reconstructions for *ICECREAM* and other baseline methods; see Fig. 6 ▸. Since the volumes contain lamellae with varying thickness and tilt angles, the FSC was computed on the central subtomogram of size 256 × 256 × 256.

As with the in-distribution experiments, the FSC curve is consistently higher for *ICECREAM* than for the baselines. It is worse than in Fig. 3 ▸, where the models were trained and evaluated on the same data, but the two experiments involve different datasets; on EMPIAR-11830 the FSC for FBP reconstruction is also worse.

The FSC gain obtained by using *cryoCARE*+*IsoNet* or *DeepDeWedge* instead of FBP is smaller for this experiment, which we again attribute to the fact that the models were not trained on this specific dataset. *ICECREAM* consistently improves over all baselines.

#### 3.4.1. Warm start to accelerate processing

The performance of self-supervised models can degrade significantly when a pre-trained model is applied directly to a new dataset, especially if it is different from the training data. We now show that such a pre-trained model is nonetheless valuable as a warm start.

**Figure 7:**
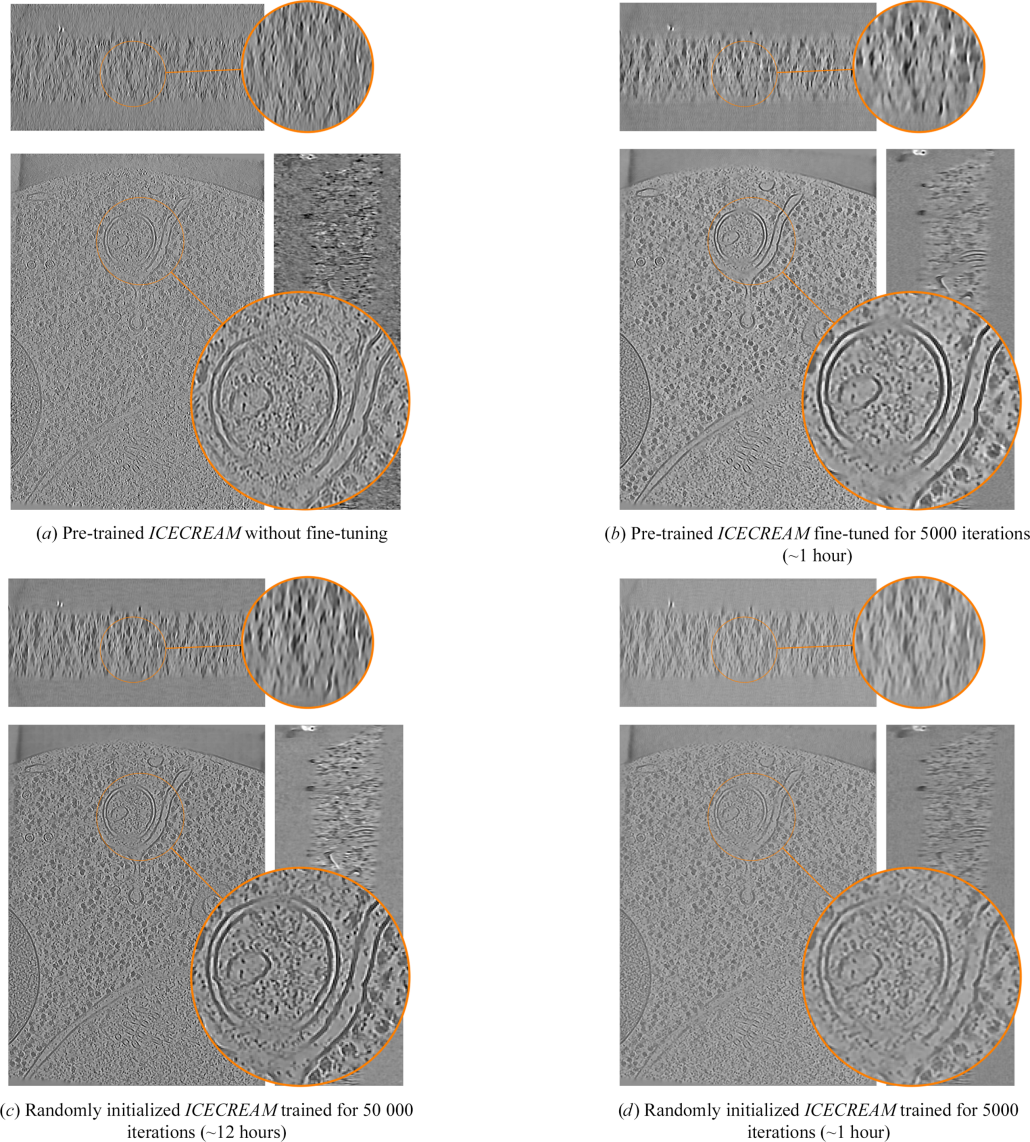
Inference of *ICECREAM* can be accelerated by fine-tuning a pre-trained network, reducing the runtime from 9 to 1 h. The models have been applied to a tomogram from EMPIAR-14162.

We used the neural network trained on the ten volumes of the EMPIAR-11830 dataset as the pre-trained model and fine-tuned it for only 5000 iterations. Fig. 7 ▸ shows the orthogonal slices of the reconstruction using only the pre-trained network, the network warm-started at the pre-trained weights and the network trained from random initialization for 50 000 iterations or about 12 h.

**Figure 8:**
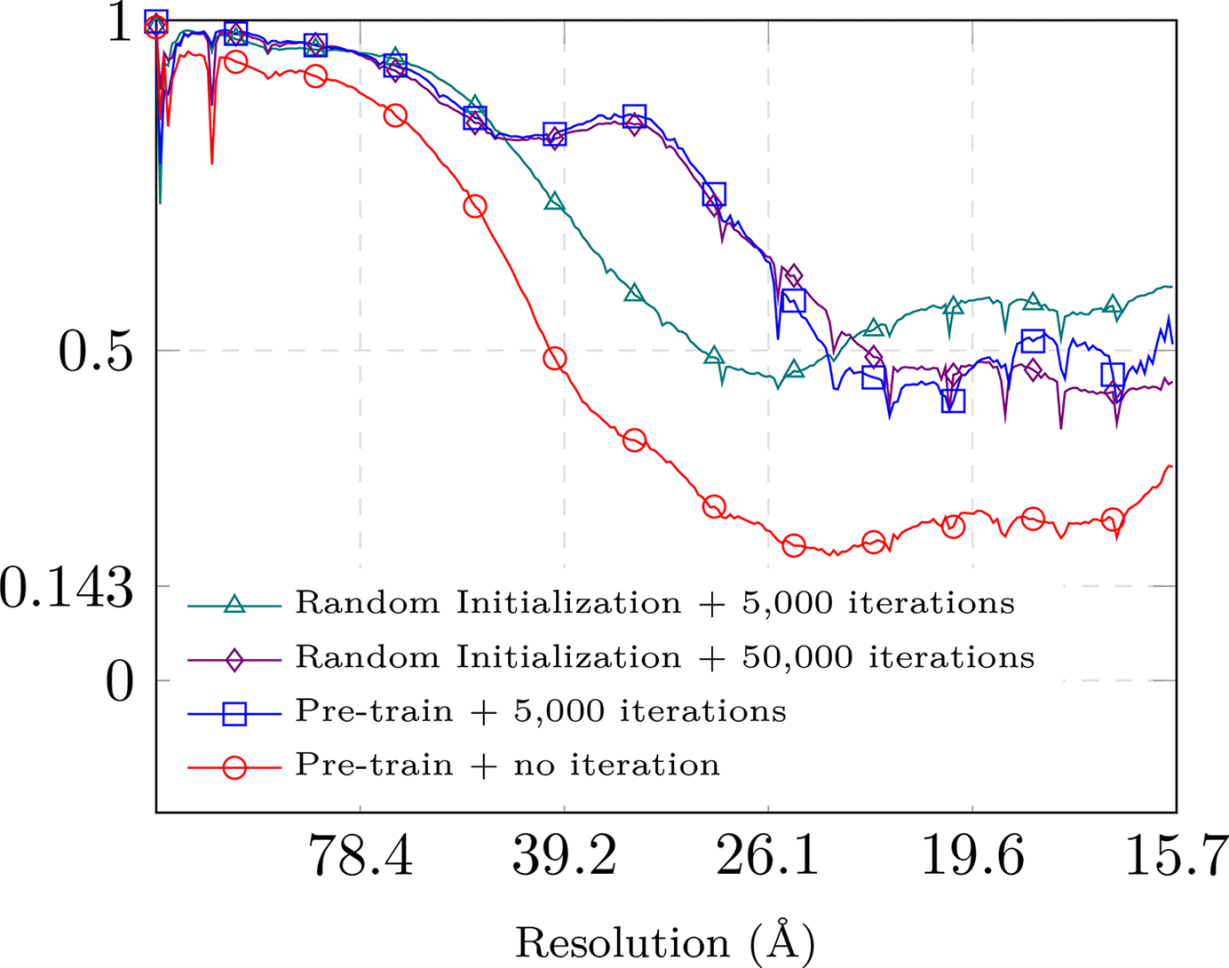
Influence of different fine-tuning strategies on the FSC. The reference volume is obtained by training the pre-trained model for 50 000 iterations. The FSC curves are computed using the final reconstructions of the respective models obtained after training for 50 000 iterations as a reference.

**Figure 9:**
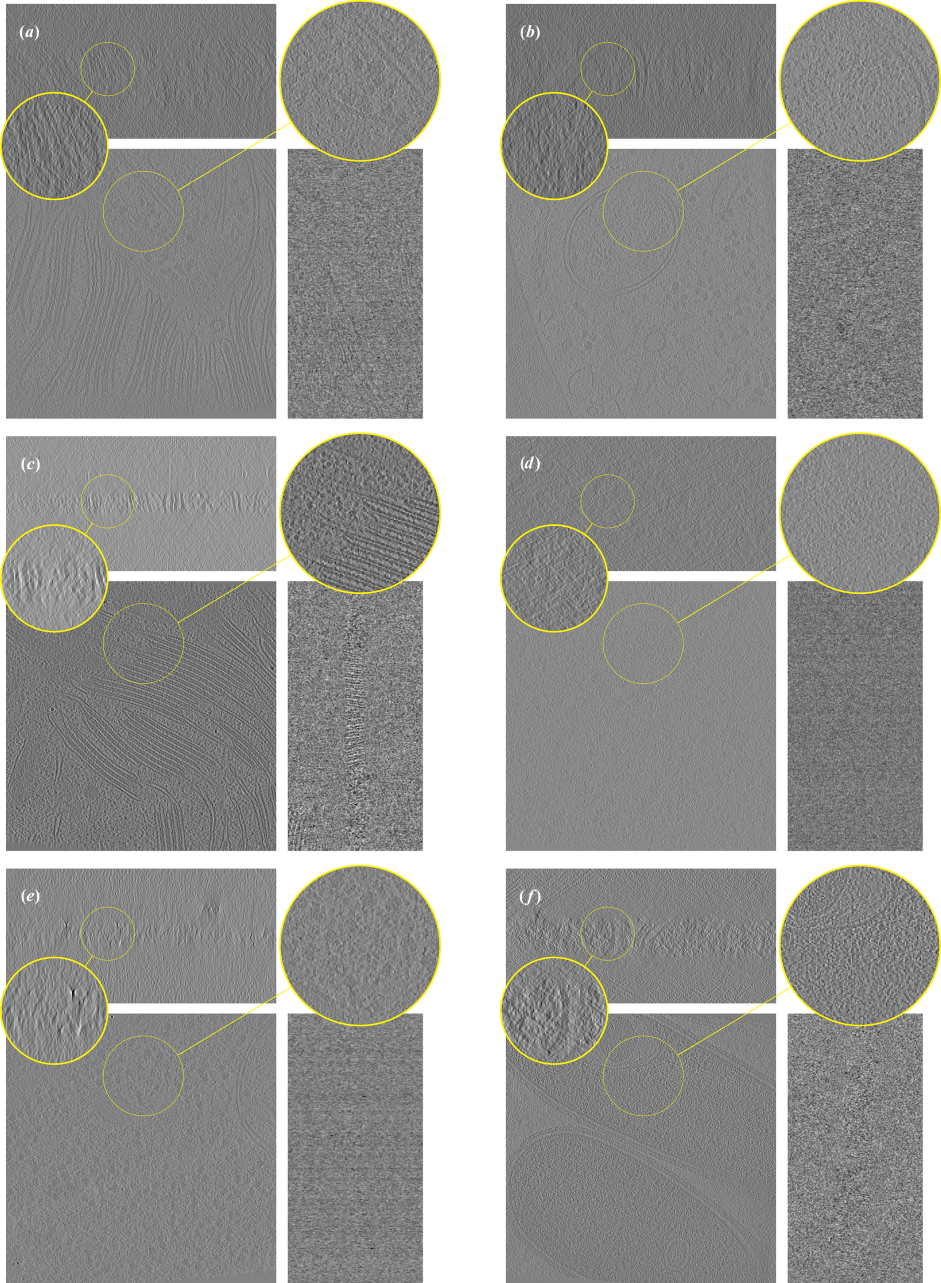
FBP reconstruction of the tomograms reconstructed in Fig. 1 ▸.

**Figure 10:**
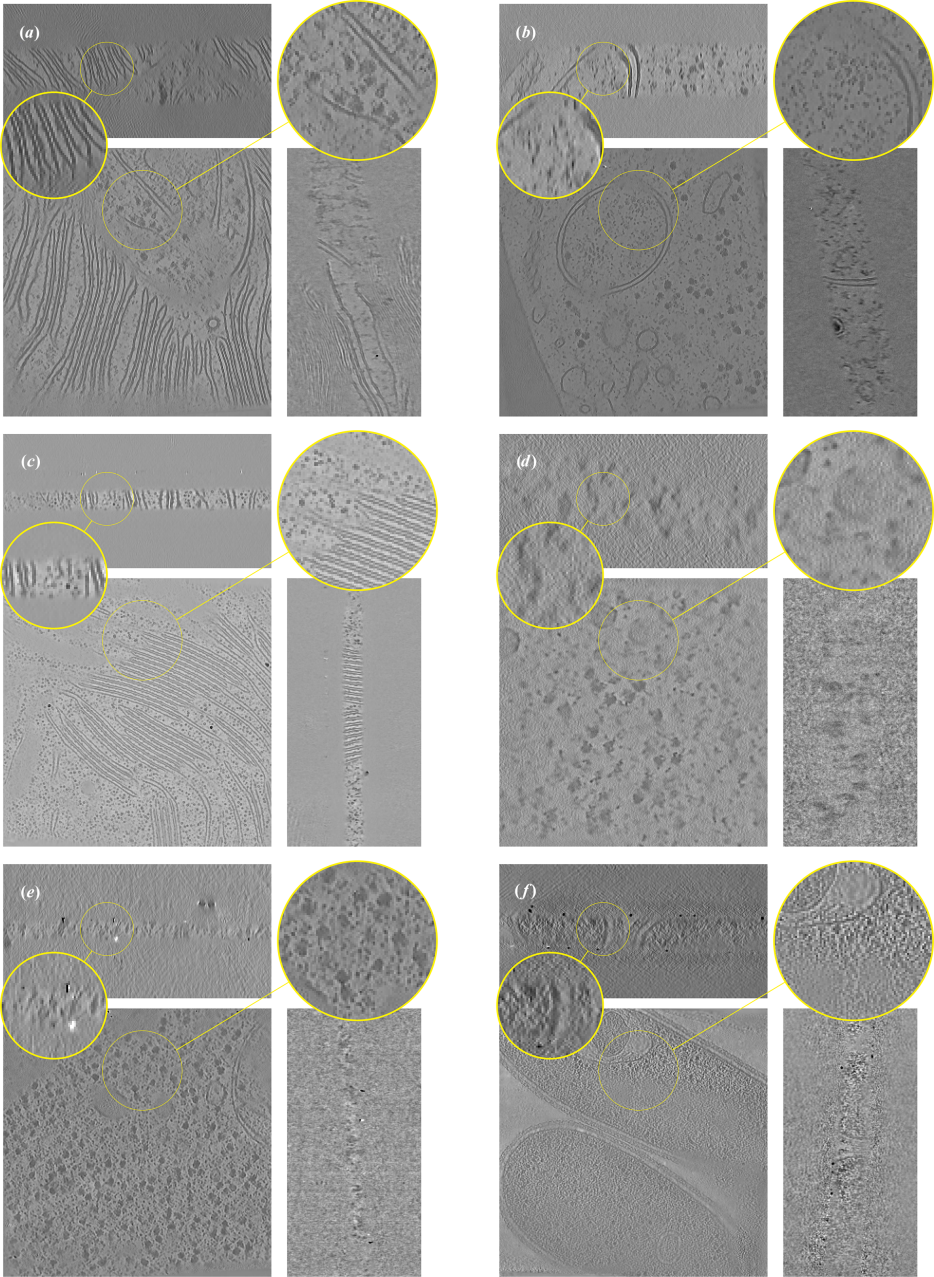
*DeepDeWedge* reconstructions of the tomograms used in Fig. 1 ▸.

**Figure 12:**
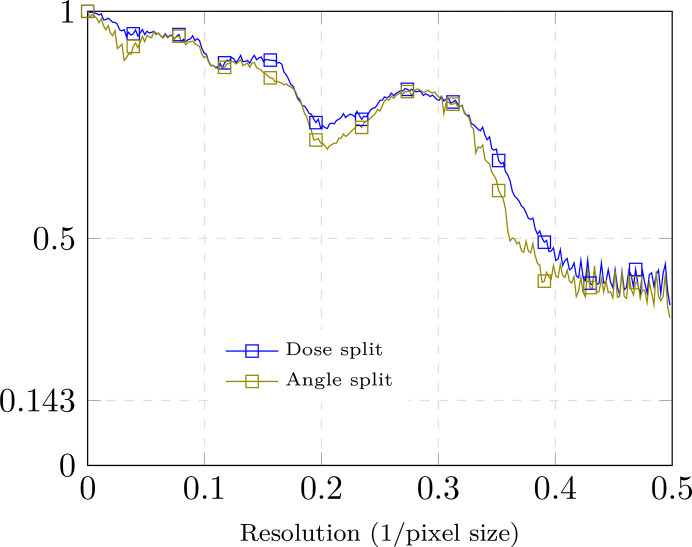
Dose versus tilt splitting for *ICECREAM* on the dataset of *C. reinhardtii* flagella.

It can be seen that simply using the pre-trained network gives a noisy reconstruction with missing details. Remarkably, however, fine-tuning the pre-trained model for a mere 5000 iterations (or about an hour) performs similarly to the randomly initialized network trained for 50 000 iterations. While training a randomly initialized network for 5000 iterations improves over the direct application of the pre-trained model, it does not reach the fine-tuning FSC. We further observe that the behavior of the FSC can be hard to interpret at high frequencies as training a randomly initialized network for 5000 iterations yields a higher FSC than other approaches. We attribute this to the fact that excessive suppression of high-resolution data can lead to spuriously high FSC values. These observations are quantitatively validated by the FSC in Fig. 8 ▸. All FSC curves are computed using the final reconstructions of the respective models obtained after training for 50 000 iterations as a reference.

## 4. Discussion and conclusion

We showed that self-supervised learning grounded in equivariance coupled with careful engineering can attain state-of-the-art empirical performance in cryo-ET reconstruction. This puts forth a number of interesting theoretical and applied questions.

For example, *ICECREAM* assumes that the distribution of subtomograms is invariant under (a subgroup of) rotations. In practice this holds only approximately, so that even with large datasets, expressive networks and global optimization, the minimizer of the equivariant loss would not recover the estimator that could be computed with access to ground truth. It is therefore important to quantify (i) the deviations of real volumes from rotational invariance and (ii) how learning proceeds under this mismatch, especially because the equivariant loss alone, without data consistency, admits trivial minimizers arising from the double application of *f*_ϕ_. The importance of this analysis is amplified by the fact that deep models can hallucinate structure. Potential biologically salient findings should thus be corroborated with independent methods, including the noisy but bullet-proof FBP.

Training time remains a limitation. *ICECREAM* is still best when trained per tomogram. Techniques from the deep-learning literature, such as meta-learning (Tancik *et al.*, 2021 ▸; Zhang *et al.*, 2022 ▸) and low-rank adapters (Hu *et al.*, 2021 ▸), may help speed this up. We already showed that a pretraining–fine-tuning strategy lowers the reconstruction time by roughly an order of magnitude. Further exploration of cryo-ET foundation models, large pretrained backbones adapted with lightweight heads, seems to be a promising direction (Liu *et al.*, 2024 ▸; Subramanian *et al.*, 2023 ▸; Pyzer-Knapp *et al.*, 2025 ▸).

One immediate possibility is to use *ICECREAM* reconstructions to curate training data for supervised methods such as *CryoLithe* (Kishore *et al.*, 2025 ▸), which are much faster at inference. In practice one might switch between the two strategies depending on the type of analysis and the observed performance.

We designed *ICECREAM* to be easy to use by practitioners who are not specialists in training deep neural networks. It requires only a minimal configuration and set of dependencies to obtain high-quality reconstructions. Finally, the approach presented here is not restricted to biological imaging. Electron tomography in materials science also suffers from the missing-wedge problem (often with higher SNR; Midgley & Dunin-Borkowski, 2009 ▸); in such cases, it should be possible to use *ICECREAM* without much modification.

## Figures and Tables

**Figure 11:**
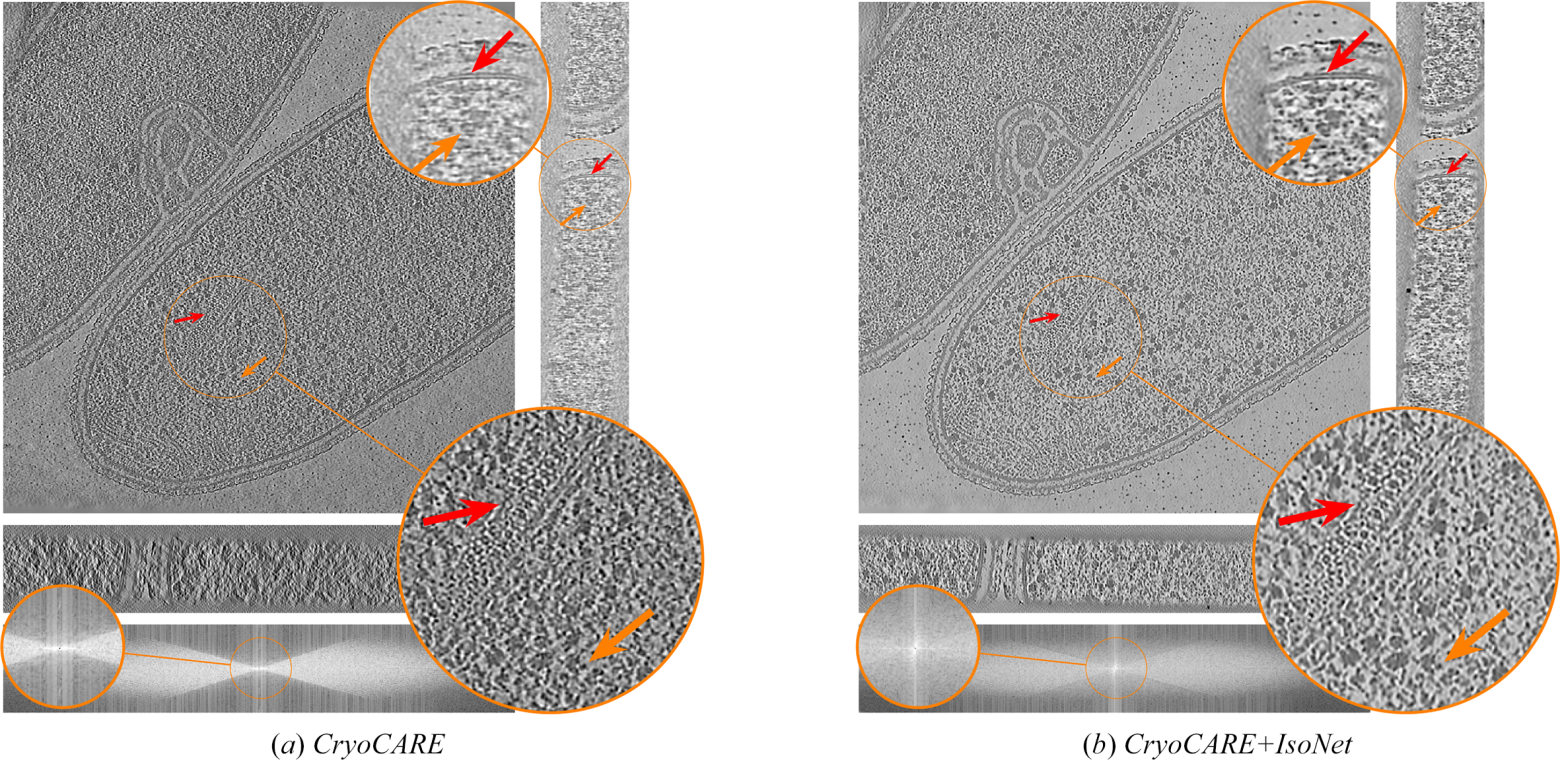
Orthogonal slices of the *T. kivui* (EMPIAR-11058) reconstruction using state-of-the art denoising and missing-wedge correction methods. Baseline methods are displayed in Fig. 2 ▸.

**Figure 13:**
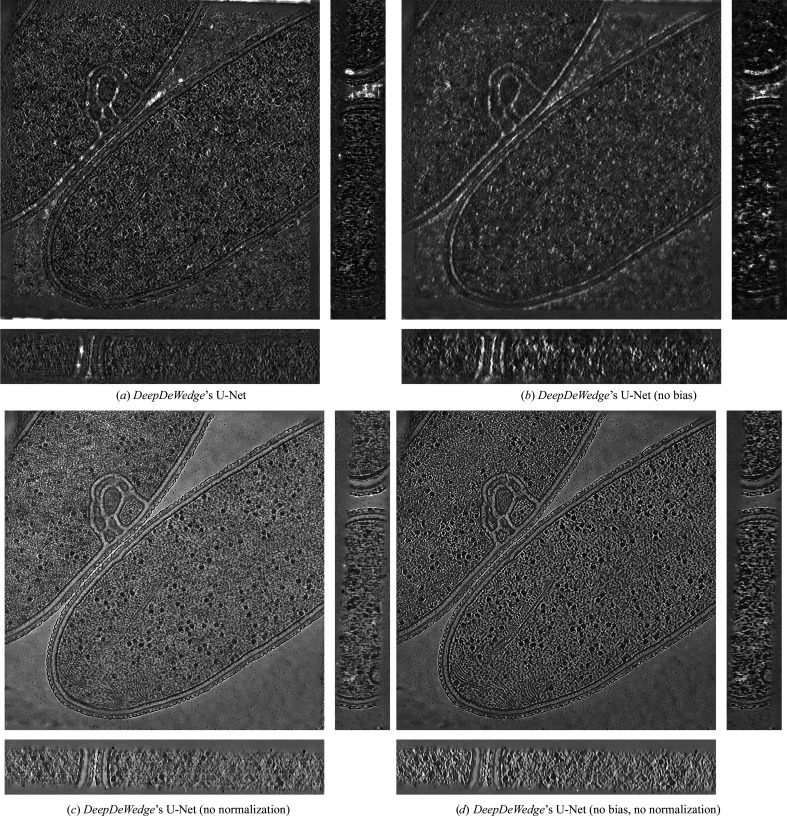
Reconstructions using *ICECREAM* with various neural network architectures. Results are obtained on the *T. kivui* dataset.

**Figure 14:**
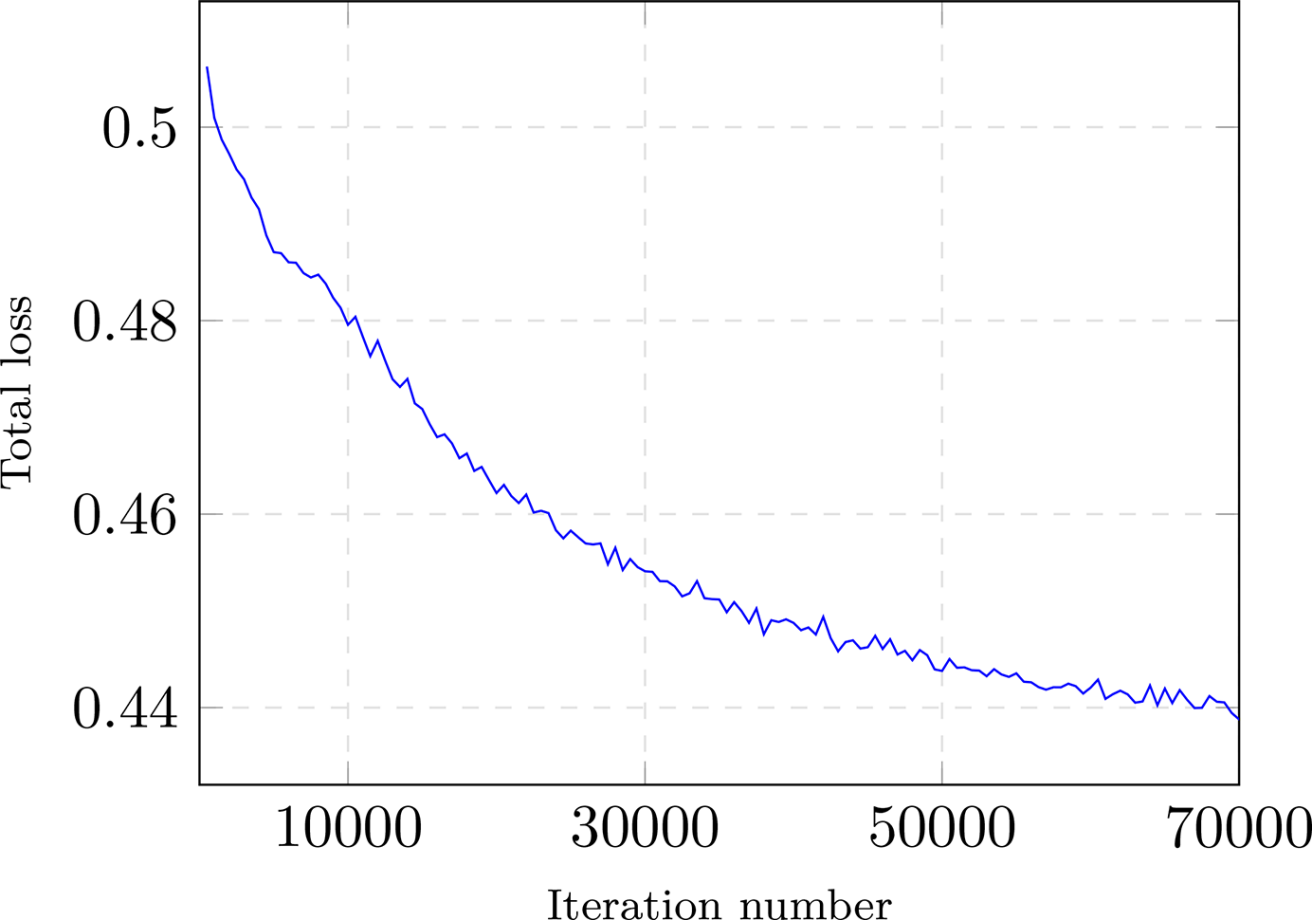
Training loss over a large number of iterations with the *T. kivui* data (EMPIAR-11058).

**Figure 15:**
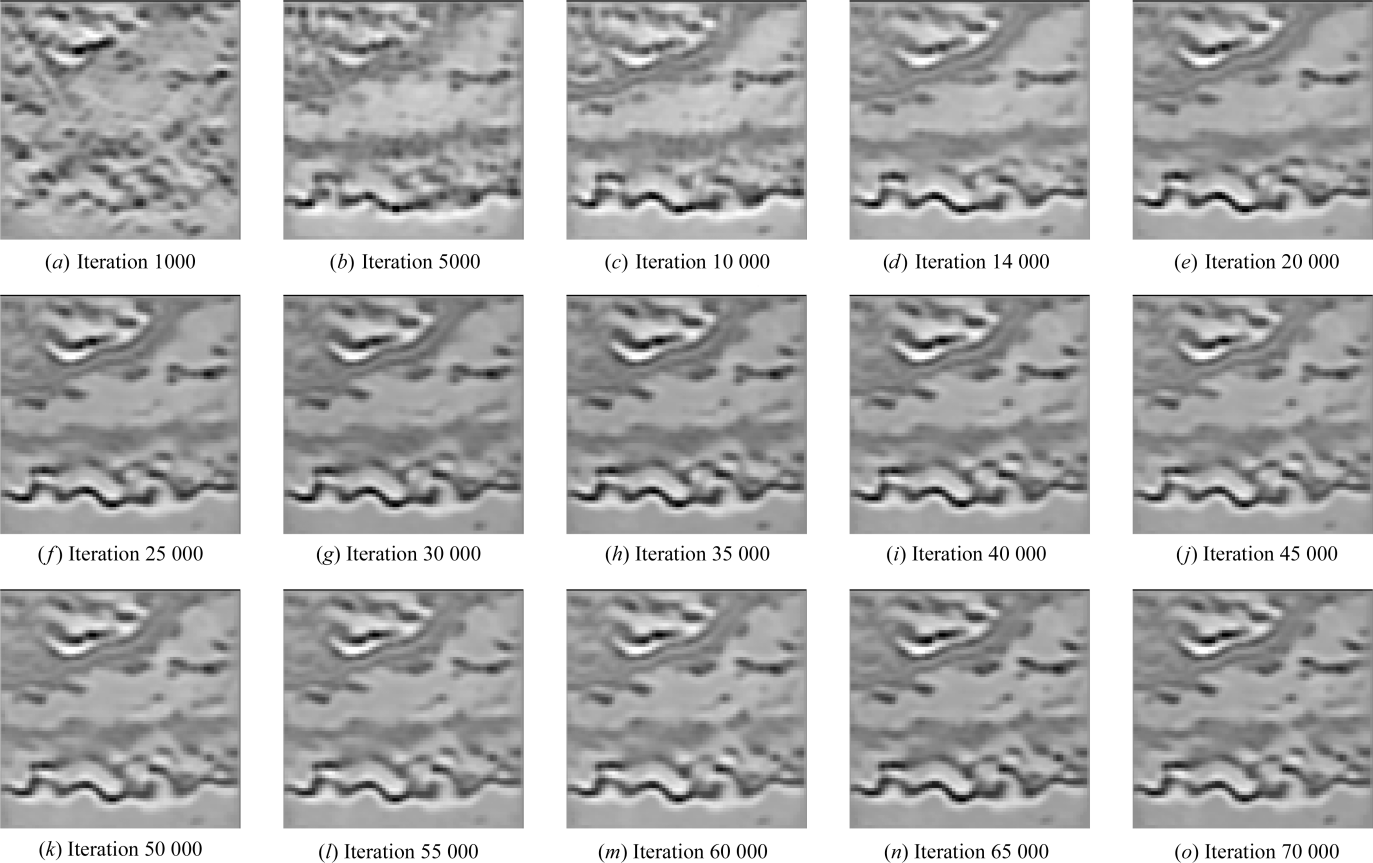
Crops, from the training set, at different training times. We quickly see only minor changes.

**Figure 16:**
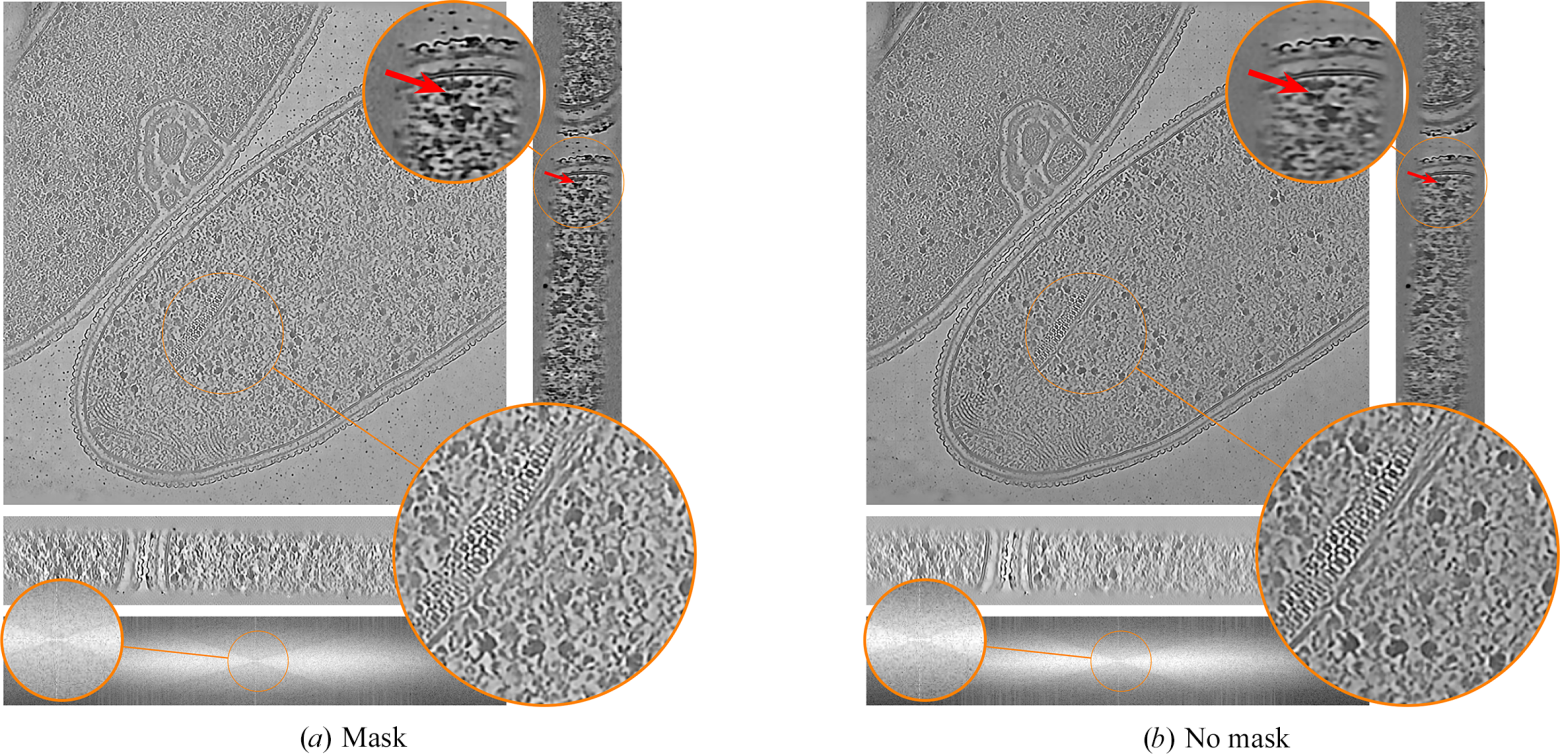
Influence of masking the original wedge in the equivariant loss. Using a mask in the equivariant loss results in a visually slightly more detailed reconstruction, as shown by the red arrow.

**Table 1:** Information about the tilt series used to generate Figs. 1 ▸, 9 ▸ and 10 ▸ The reported pixel size is after the tomograms have been downsampled by a factor of 4.

Image	EMPIAR ID	Tomogram	Pixel size (Å)	Dimensions (pixels)	Split type
(*a*)	11830	9	7.84	1024 × 1024 × 512	Dose
(*b*)	11830	2	7.84	1024 × 1024 × 512	Dose
(*c*)	12612	38	14.08	928 × 928 × 464	Dose
(*d*)	11538	1435	4.0	1024 × 1024 × 512	Angle
(*e*)	11658	1	7.84	1024 × 1024 × 512	Angle
(*f*)	11058	3	14.08	928 × 928 × 464	Angle

## Footnotes

1 The name *filtered backprojection* comes from the real-space implementation, which involves a high-pass filtering of the projection stack, followed by a back-projection which estimates the value of a voxel by summing (integrating) over the (filtered) lines passing through it. We describe the Fourier-space implementation which is simpler and more important for our exposition.

2 This is again an approximation, since FBP introduces long-range correlations in noise.

3 Since *R*_*g*_ is orthogonal, in the practical implementation we use the fact that .

## Appendix A Differences between *ICECREAM* and existing self-supervised algorithms

The proposed approach is closely related to existing self-supervised methods for cryo-ET, namely *DeepDeWedge* and *IsoNet*. In the following, we describe these approaches and highlight the differences which lead to *ICECREAM*’s higher quality reconstructions.

### A1. DeepDeWedge

*DeepDeWedge* uses a loss function similar to that used by *ICECREAM*, a variant of equivariance loss, but with several key differences. The two methods also use rather different pre-processing steps.

*DeepDeWedge* starts by pre-processing the full tomogram to correct for the contrast transfer function (CTF) using *IsoNet*’s CTF deconvolution routine. It then extracts overlapping cubic subtomogram pairs  and normalizes them by batch to generate two subtomograms: the input of the model *v*_0,*g*_ = *A*[*R*_*g*_(*y*′_0_)], which is rotated and has a ‘manually’ removed missing wedge, and *v*_1,*g*_ = *R*_*g*_(*y*′_1_), the rotated target. Note that unlike the finite set of 40 rotations used earlier, *DeepDeWedge* uses arbitrary 3D rotations from the rotation group [*g* ∈ SO(3)].

#### A1.1. Differences in the loss function

The *DeepDeWedge* loss is

Differently from *ICECREAM*, *DeepDeWedge* uses a particular weighting applied in Fourier space. The first term of the loss computes the equivariance loss with a stronger weight only on the span of (*I* − *A*)*R*_*g*_*A*_*g*_, which is the (unrotated) missing wedge without the ‘manually’ introduced one in case they overlap. The equivariance loss is evaluated with a lower weight on the visible wedge (similarly corrected for the ‘manual’ one). Importantly, the equivariance loss in *DeepDeWedge* is different from that used in *ICECREAM* in that it only applies *f*_ϕ_ once in the visible wedge. As a consequence, *DeepDeWedge* only uses one pass of the network at inference, while *ICECREAM* requires two passes, which we believe explains the improved quality of the denoising.

In *ICECREAM*, we evaluate the loss with the same weighting everywhere. Further, the *g*-rotated missing wedge is absent; see equation (5). This allows the regularization by equivariance to act everywhere in Fourier space. The importance of denoising in the visible wedge is controlled by the data-fidelity loss (equation 4).

#### A1.2. Iterative subvolume updates

Similarly as in *IsoNet*, *DeepDeWedge* training uses a form of boosting: the entire training set of subvolumes is periodically updated using the current trained network and then treated as a new training set. As in *IsoNet*, this is performed only on the missing wedge, while keeping the observed wedge untouched (and thus noisy).

*ICECREAM* is simply trained on the dataset by minimizing a principled loss, without boosting. This is motivated by the framework of equivariant imaging, which suggests using the clean volume (Chen *et al.*, 2021 ▸). Since the nondegraded volume is not available, we substitute the output of the network at the current iteration. The double application of the network in the equivariant loss plays a similar role as boosting in *DeepDeWedge* and *IsoNet*, but in a principled way which avoids the drawbacks of boosting. As explained in Section 2.7, it also means that we evaluate the network twice at inference. As explained in the previous paragraph, strong denoising is achieved in *ICECREAM* with the data-fidelity loss in equation (4) which is not present in *DeepDeWedge*. The regularization enforced by the data-fidelity loss is fundamentally different to that imposed by the equivariance loss (which rotates the sub­tomograms) and is similar to *cryoCARE* denoising (Buchholz *et al.*, 2019 ▸).

#### A1.3. Scaling-equivariant architecture

Similarly as in *DeepDeWedge*, we use an architecture inspired by the original U-Net. A key difference is that we design the network to be scaling equivariant: we use the 3D U-Net from Wolny *et al.* (2020 ▸), but with biases removed from all convolutional layers and without normalization layers. This is very important in our setting: iterated application of the network to subtomograms changes spatial statistics between passes; see Appendix *E*[App appe] for a related ablation. It has been shown by Mohan *et al.* (2020 ▸) that scale-equivariant networks are robust, interpretable and generalize well to unseen noise characteristics.

### A2. IsoNet

*IsoNet* differs more substantially from *ICECREAM* and *DeepDeWedge*, but it shares the key characteristic that the dataset is augmented by rotating the subtomograms and removing the wedge. Similar to *DeepDeWedge*, the first step of *IsoNet* is to pre-process the input tomogram by compensating for the CTF. Unlike *DeepDeWedge* (but similarly to *ICECREAM*), subtomograms are extracted using a criterion that favors non-empty regions. At each iteration the sub­tomograms are rotated by randomly sampling among 20 rotations that require no interpolation; we adopt this strategy in *ICECREAM*.

An important advantage of *IsoNet* is that it does not require two observations of the same structure. This is circumvented by adding noise to the input of the network before removing the missing wedge. If *v* is the observed subtomogram, *IsoNet*’s loss function is

where η is Gaussian noise. The subtomogram *v* is iteratively updated by using the current neural network, *v* ← *f*_ϕ_(*v*). *IsoNet*’s loss is similar to the equivariance loss defined in equation (5) as modulo noise and the fact that it is evaluated on the entire Fourier space, including the original missing wedge. In practice, *IsoNet* is often combined with *cryoCARE* to boost denoising. In *ICECREAM*, the denoising step is directly included in the data-fidelity loss (equation 4).

## Appendix B Self-supervised versus supervised denoising

In self-supervised learning we do not have access to ground-truth training targets. It is nonetheless possible to establish certain equivalences between the supervised and self-supervised losses by exploiting statistical independence or symmetries of data and physics. For example, under our assumption that the measurements  are independent and that

one can show that the minimizer of the expected data-fidelity loss is equal to the minimizer of its supervised counterpart,

where the expectation is over *y*_0_. Indeed, following the standard derivation (see, for example, Lehtinen *et al.*, 2018 ▸; Wiedemann & Heckel, 2024 ▸), we have

Taking expectation over *y*_0_ and using , we obtain

The only term depending on the optimization variable ϕ is , which is the supervised data-fidelity loss under the observation of *y*_1_.

## Appendix C Reconstruction of diverse cryo-ET datasets

We display the reconstruction of the volumes displayed in Fig. 1 ▸ when using FBP reconstruction (Fig. 9 ▸) and *DeepDeWedge* reconstruction (Fig. 10 ▸).

For completeness with respect to Fig. 2 ▸, we display the reconstruction of the *T. kivui* dataset using *cryoCARE* and *cryoCARE*+*IsoNet* in Fig. 11 ▸.

## Appendix D Dose versus tilt splitting

We compare the FSC curves of reconstructions obtained with *ICECREAM* when splitting the dose versus splitting the tilts (Fig. 12 ▸). Even though the difference is small, dose splitting seems to produce better reconstructions in terms of FSC.

## Appendix E Architectural experiments

We experiment using the U-Net architecture from *DeepDeWedge* in the *ICECREAM* method. Notice that the U-Net present in *DeepDeWedge* is not scale equivariant, as opposed to the architecture of *ICECREAM*. We observed that removing learnable biases and instance normalization layers used in *DeepDeWedge*’s U-Net implementation improves the reconstruction obtained when used in *ICECREAM*. We use the *T. kivui* dataset where all the training parameters are kept constant except for the model architecture. Fig. 13 ▸ shows reconstructions using *ICECREAM* with various neural network architectures. We observed that removing normalization leads to the most noticeable improvement. Additionally, removing the biases further improves the quality of the reconstruction.

## Appendix F Ablation studies

### f1. When to stop training?

A standard approach when training deep neural networks is to set aside a subset of the volume as a validation dataset and to compute the loss on this held-out data. However, this strategy presents two main drawbacks in our setting. Firstly, obtaining a reliable validation loss requires computing it frequently and averaging over several iterations, which would significantly increase the training time, which is already the main limiting factor. Secondly, the number of available subvolumes per dataset is limited, and excluding a non-negligible fraction (for example 10%) from training may degrade the final performance of the model. For these reasons, we chose to not rely on the validation loss.

Instead, we propose to rely directly on the training loss, which often serves as a reasonable proxy for monitoring the training dynamics (see Fig. 14 ▸). More importantly, we periodically save the reconstruction of a fixed crop used during training. This qualitative monitoring proves particularly effective for determining when training has reached a satisfactory stage. In our experience, the reconstructed crop rapidly attains a visually coherent state and exhibits only marginal improvements with further training (see Fig. 15 ▸). Figs. 14 ▸ and 15 ▸ are obtained while training *ICECREAM* on *T. kivui* data (EMPIAR-11058).

### f2. Influence of masking the original missing wedge

The equivariant loss requires two applications of the neural network. The first one serves as a pre-filling of the missing wedge to obtain something more realistic than a zero-filled missing wedge. This intermediate reconstruction is then rotated, and the neural network is trained to fill a new missing wedge on the artificially undegraded input.

As the original missing wedge is filled by the network in order to create a more natural volume, there is no guarantee that the filled missing wedge is physically admissible. Therefore, we do not necessarily want the network to learn to reproduce such a wedge, and mask it in the equivariant loss (equation 5). However, in the original equivariant imaging framework (Chen *et al.*, 2021 ▸) there is no such mask. We therefore visually analyze the impact of using such a mask or not. Using the masking operator *A*_*g*_ corresponds to using the equivariant loss (equation 5), and not using the masking as using the following loss

We report the visual results in Fig. 16 ▸ on *T. kivui* data (EMPIAR-11058). We observe very little difference, only a small advantage of using the mask, where tiny structures and high-contrast features appear slightly less blurred, especially along the *z* axis.
